# Supplementary material for: Optical Flow Sensor with Fluorescent-Conjugated Hyperelastic Pillar: A Biomimetic Approach
Source: Biomimetics (Basel). 2024 Nov 22;9(12):721. doi: 10.3390/biomimetics9120721 (PMC11673239; doi:10.3390/biomimetics9120721)
Supplement: Supplementary file 1 [file biomimetics-09-00721-s001.zip › biomimetics-3309171-supplementary.pdf]

Supplemental Information

Optical Flow Sensor with Fluorescent-Conjugated Hyperelastic Pillar: A Biomimetic Approach

Dongmin Seo [1], Seungmin Yoon [2], Jaemin Park [2], Sangwon Lee [2], Seungoh Han [3], Sung-Hoon Byun [4] and Sangwoo Oh [4,*]

[1] Department of Electrical and Electronic Engineering, Semyung University, Jecheon 27136, Republic of Korea; dseo@semyung.ac.kr
[2] Department of Electrical Engineering, Semyung University, Jecheon 27136, Republic of Korea; ysmin5792@gmail.com (S.Y.); jm77788828@gmail.com (J.P.); sw2796@naver.com (S.L.)
[3] Department of Robotics, Hoseo University, Asan 31499, Republic of Korea; sohan@hoseo.edu
[4] Ocean and Maritime Digital Technology Research Division, Korea Research Institute of Ships and Ocean Engineering, Daejeon 34103, Republic of Korea; byunsh@kriso.re.kr
* Correspondence: swoh@kriso.re.kr; Tel.: +82-42-866-3615; Fax: +82-42-866-3624

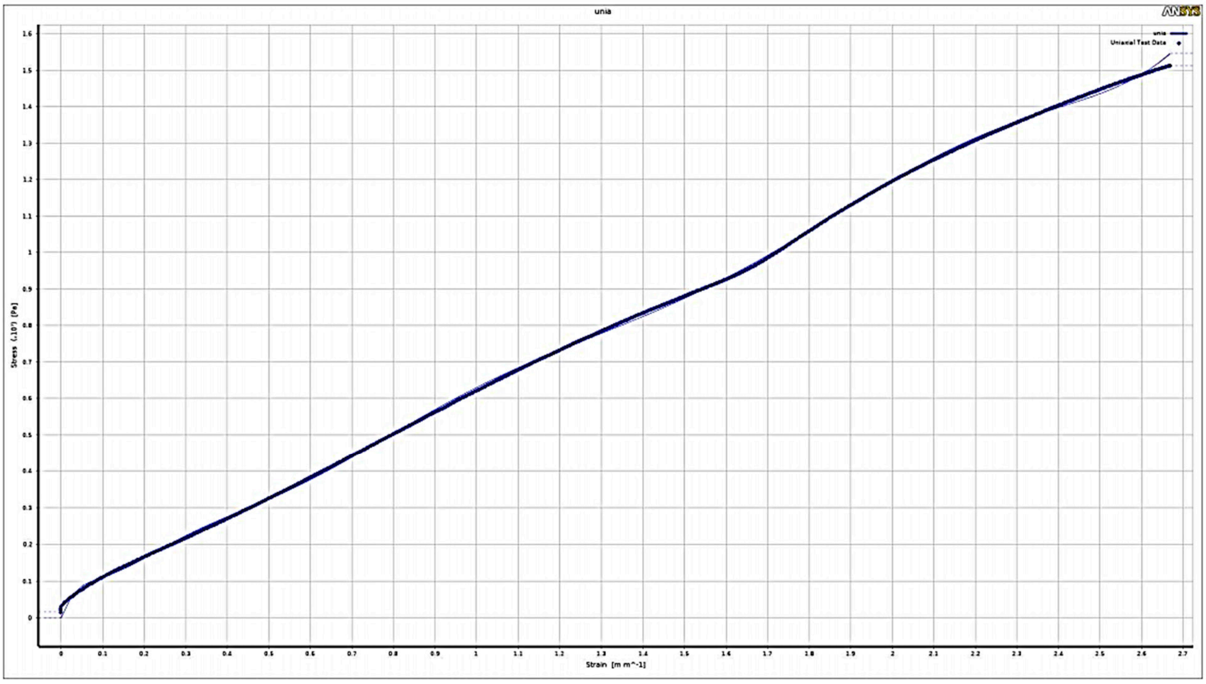

Figure S1. Results of fitting values derived from tensile test to Mooney–Rivlin nine-parameter model.

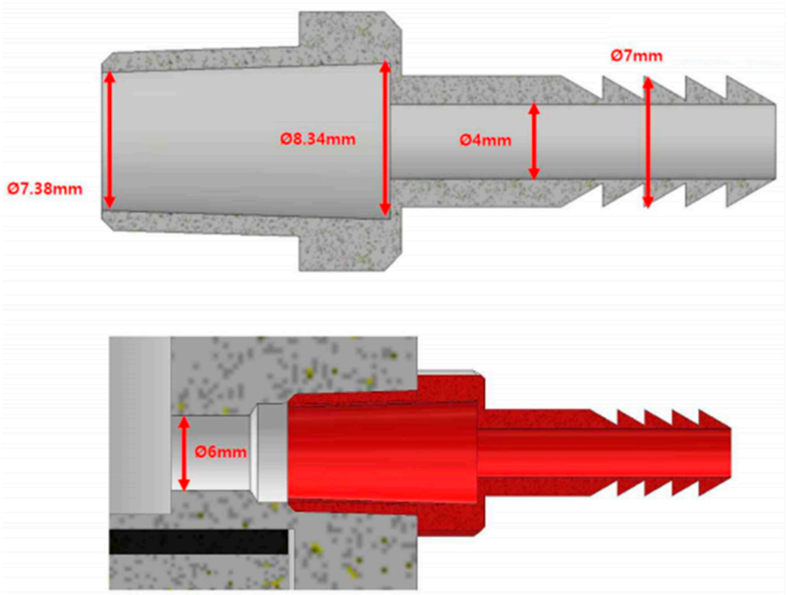

Figure S2. Detailed schematic diagram of nozzle.

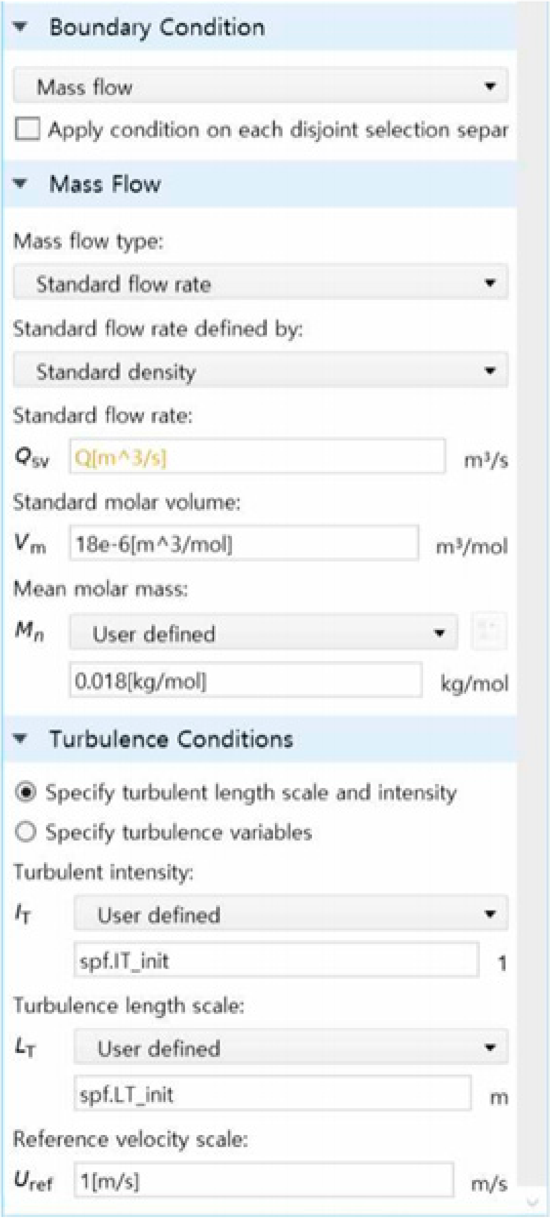

Figure S3. Conditions of boundary layer for flow field within chamber.
